# Mate-choice for close kin is associated with improved offspring survival in *Lodoicea maldivica*, the largest-seeded plant in the world

**DOI:** 10.1038/s41598-023-41419-4

**Published:** 2023-09-18

**Authors:** Emma J. Morgan, Christopher N. Kaiser-Bunbury, Peter J. Edwards, Frauke Fleischer-Dogley, Chris J. Kettle

**Affiliations:** 1 ITES–Ecosystem Management, ETH Zürich, Universitätstrasse 16, 8092 Zurich, Switzerland; 2 Centre for Ecology and Conservation, College of Life and Environmental Sciences, University of Exeter, Cornwall Campus, Penryn, UK; 3 IBZ–Institute of Integrative Biology, ETH Zürich, Universitätstrasse 16, 8092 Zurich, Switzerland; 4 Seychelles Islands Foundation, Victoria, Mahé Seychelles; 5 Bioversity International, Via di San Domenico 1, 00153 Rome, Italy

**Keywords:** Ecological genetics, Evolutionary ecology

## Abstract

We studied spatial patterns of kinship in the offspring of the endangered *Lodoicea maldivica,* a dioecious palm that produces the largest seed of any plant. Previous research has suggested that restricted seed and pollen dispersal in populations resulted in strong spatial genetic structure. We used microsatellites to genotype young plants and their potential parents at four sites across the species’ entire natural range. We determined the most likely parents of each young plant based on the spatial separation of each parent pair, their genetic relatedness, and the level of correlated paternity. We identified both parents (43 female, 54 male) for 139 of 493 young plants. Mean distance between parental pairs was 26.8 m. Correlated paternity was low (0.168), indicating that mother trees were often pollinated by several fathers. Parental pairs were more closely related than expected by chance, suggesting outbreeding depression. Our results highlight the apparent strong mate choice for close kin in parent pairs of surviving offspring. We discuss the alternative biological processes that could lead to this, including the potential for break-up of favourable allelic combinations necessary for the development of the palm’s very large seed. Management implications include germinating seeds where they naturally fall, using a diverse range of male plants as pollen donors for hand pollination, and protecting the native community of gecko pollinators.

## Introduction

The genetic relatedness of mating pairs has long been understood as a critical factor influencing offspring vigour. It is often found that mating between closely related individuals leads to reduced fitness or inbreeding depression^1^, which can be explained both by the loss of the heterozygote advantage or heterosis and by the negative effects of homozygosity of partially recessive detrimental mutations^2,3^. Species vary, however, in their vulnerability to inbreeding depression; plants with mixed mating systems, for example, are usually less affected than outbreeding species^4^. Also, species that occur naturally in small or isolated populations may be less susceptible to negative effects of inbreeding, presumably because deleterious alleles have been purged from their populations over many generations^5^. The effectiveness of purging remains unclear, however, and inbred populations may accumulate genetic load gradually, with negative consequences for their long-term fitness^1,6^.

Compared to inbreeding, matings between less related individuals usually exhibit greater fitness^2^. In some cases, however, outbreeding results in lower fitness, especially when the two individuals come from different populations. Possible genetic reasons for such outbreeding depression include adaptive differentiation among populations, fixed chromosomal differences between populations, and the existence of coadapted gene complexes^7–9^. Whereas the first two factors are most likely to appear in crosses between widely separated or ecologically distinct populations or sub-species (e.g.^10^), the break-up of coadapted gene complexes may also occur at a small spatial scale within populations^11^. For example, several experimental studies have shown that crossing distance within a site can significantly affect the fitness of progeny, sometimes with inbreeding depression at short distances and outbreeding depression at large distances. When the herb *Delphinium nelsonii* was hand-pollinated across different distances within a population^11^, progeny from the intermediate crossing distances (3 and 10 m) grew approximately twice as large as the more inbred (1 m) and outbred (30 m) progeny. Similarly, both inbreeding and outbreeding depression were detected in an experimental study of the Australian shrub *Grevillea mucronulata*, with all measures of fitness (seed set, seed size, germination and seedling growth) being higher for pollen transfers across intermediate compared to maximum and minimum distances^12^.

The effects of inbreeding depression have become a major concern for plant conservation, as the distributions of many species become increasingly fragmented. This concern has prompted numerous efforts to restore ecological connectivity in the landscape and also to promote outbreeding by increasing the genetic diversity of small populations^5^. Introducing distant genotypes into a threatened population is not without risk, however, since it could lead to outbreeding depression^2,13,14^. A particular problem in screening for outbreeding depression, especially in long-lived, slow-growing species, is that any fitness reductions may be delayed until the F_2_ or later generations, due to the time taken for deleterious interactions to become exposed^2^.

We studied possible inbreeding and outbreeding depression in populations of the endangered coco de mer palm *Lodoicea maldivica* (hereafter *Lodoicea*). This dioecious palm is best known for its huge seed—by far the largest in the plant kingdom—which can weigh as much as 18 kg. The species forms dense, almost monospecific stands on two small islands, Praslin and Curieuse, in the Indian Ocean archipelago of Seychelles. In the absence of any mechanism other than gravity for dispersing fruits, the huge seedlings of *Lodoicea* typically grow in clusters around the mother plant. The very limited seed dispersal is reflected in the genetic make-up of populations, which exhibit high levels of inbreeding and a strongly developed fine-scale spatial genetic structure (FSGS)^17^.

Given the unusual features of the *Lodoicea* life history, three possibilities relating to its breeding system seem worth investigating: (1) inbreeding depression occurs due to very short seed dispersal distances and a strongly developed FSGS; (2) outbreeding depression occurs, perhaps linked to the break-up of coadapted gene complexes involved in the development of the huge seed; (3) both inbreeding and outbreeding occur, as reported in some other plant species^11,12^. To distinguish between these possibilities, we used microsatellites to characterise the genotypes of young *Lodoicea* and all possible father and mother plants growing in their vicinity. These data were then used to determine the most likely parents of each offspring plant, allowing us to calculate their spatial separation (pollination distance) and genetic relatedness (kinship coefficient). Finally, we compared the distribution of kinship coefficients for parental pairs with the expected distribution if mating was random. In this comparison, fewer closely related parental pairs than expected by random mating would indicate inbreeding depression (or avoidance), while fewer than expected distantly related parental pairs would indicate outbreeding depression. These analyses were performed separately for male and female offspring to investigate whether there were any sex-specific differences in inbreeding or genetic diversity, as reported for some dioecious species (e.g. *Pistacia atlantica*^15^; *Simmondsia chinensis*^16^. Our study also contributes to the growing body of work exploring the role of pollen dispersal in shaping patterns of FSGS.

## Materials and methods

### Study area and sampling scheme

**Figure 1:**
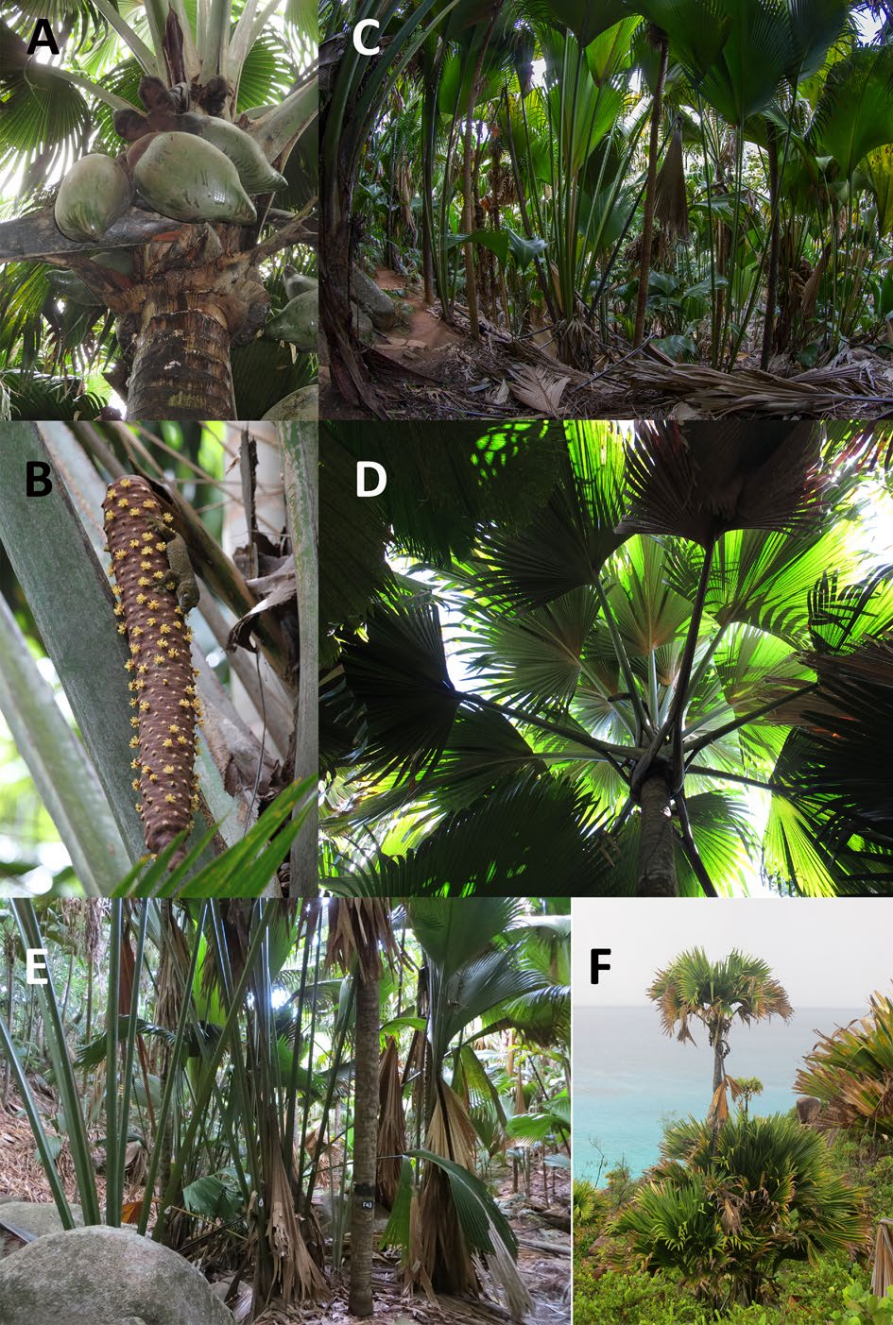
Fruits (**A**) and male inflorescence (with a gecko *Ailuronyx trachygaster*) (**B**) of the coco de mer palm *Lodoicea maldivica* growing in a dense monotypic stand in the UNESCO site Vallée de Mai (**C**). View from beneath the canopy shows large, interlocking leaf fronds (**D**). Typical mixed-age cluster of coco de mer palms in the *Lodoicea* forest (**E**) and in open, degraded habitat (**F**).

The coco de mer *Lodoicea maldivica* (Fig. 1) is an endemic palm on the islands of Praslin and Curieuse in the Seychelles archipelago in the Indian Ocean. Following settlement of the islands in the late eighteenth century, the formerly dense *Lodoicea* forests were progressively cleared, and the only semi-continuous stands on Praslin today are in the southern part of the island, notably in the Vallée de Mai World Heritage Site. On Curieuse, the species occurs mainly as isolated individuals or in small clusters^18^. We investigated pollen flow at the same four locations used to study fine-scale spatial genetic structure in *Lodoicea* populations^17^. Three of these locations, referred to here as sub-populations, were on the island of Praslin—Vallée de Mai (VM), Fond Peper (FP) and Fond Ferdinand (FF)—and one was on the island of Curieuse (CU).

In each sub-population, we selected and mapped four ‘clusters’ of young plants, referred to here as offspring, that appeared to be the product of natural regeneration and showed minimal signs of human disturbance (Fig. 1). The 16 clusters had a mean area of 904 (± 801 SD) m^2^ and contained between 34 and 193 plants of varying ages. Within each cluster, we geo-referenced (Garmin 60CSx) the locations of all potential mother trees and mapped the relative positions of younger plants using a tape measure and compass. Distances between the boundaries of adjacent clusters ranged from 13 to 360 m. We also recorded the GPS locations of all possible father trees within a radius of 80–120 m of each cluster. Leaf tissue was collected from all georeferenced plants in accordance with relevant institutional, national and international guidelines and legislation. Permits to conduct the research and export samples were granted by the Seychelles Bureau of Standards and the Ministry of Environment, Energy and Climate Change, respectively.

### Molecular genetic methods

Total genomic DNA was extracted from the silica-dried leaf tissue using the DNeasy ® 96 Plant Kit (Qiagen). Twelve nuclear microsatellite markers were designed for *Lodoicea* at ecogenics GmbH (Balgach, Switzerland), using Roche 454 sequencing. For details of the polymorphic microsatellite loci and PCR conditions see^19^. Fragment length was analysed using the internal size marker LIZ 500 HD in an ABI3730 capillary sequencer (Applied Biosystems), and scored with GeneMarker 2.6.0^20^ (Appendix 1).

#### Determining the most likely parent pairs using parentage assignment

We used the delta maximum-likelihood exclusion analysis in CERVUS 3.0^21,22^. Females and males from within the cluster were included as candidate parents. This analysis simulated 10,000 offspring, with the minimum number of typed loci set at 9, the proportion of mistyped loci set at 1%, and with 96.1% of the loci typed. The proportions of candidate mothers and fathers sampled were set at 0.8 and 0.5, respectively. The strict confidence level of 95% for the trio assignment (mother-father-offspring) was used. Combined exclusion probability for the first and second parents were 0.9961 and 0.9999, respectively. We assumed that the mother and father of each assigned juvenile were the nearest assigned female and male plants, respectively^23^.

#### Estimating kinship of parental pairs

To investigate possible outbreeding or inbreeding, we calculated kinship coefficients (Loiselle’s F^24^, using SPAGeDi^25^) of male–female pairs using the genotype data for adult plants. We then compared the kinship of parental pairs (as determined in the CERVUS 3.0 analysis) with those of a reference group comprising all possible pairings of the assigned mothers (what we consider as random mating). For this latter group, we included all male trees growing within a radius of 105 m of each mother tree, this being the maximum distance over which all living male trees were sampled.

### Tree mortality

Since young *Lodoicea* plants in closed forest grow very slowly, many parent trees would have died by the time of our study, making it impossible to determine both parents. To estimate what this proportion might have been, we used data from an unpublished study of the growth and demography of *Lodoicea* populations (Seychelles Islands Foundation; SIF). From the growth rate data in that study, we estimated that our offspring plants ranged between 7 years for the youngest seedlings (trunkless plants with one or two leaves) to around 60 years for the oldest immature plants (young trunked individuals without flowers), with an average of about 44 years. Adding 7 years for the time it takes for a seed to mature, we obtained an estimate of 51 years as the average interval between pollination and time of sampling. The same study also estimated the average reproductive lifespan of *Lodoicea* (i.e., the period from reaching maturity until death) as about 100 years for male trees. On the basis of these estimates, we conclude that roughly half of all father trees would have died by the time of our study.

### Data analysis

Using pooled data for the four sites, we calculated separate linear regressions of kinship against distance (square-root transformed) for non-parental and parental pairs. We then performed an analysis of covariance (ANCOVA) to test how kinship was affected by pair type, whilst controlling for distance and site. This analysis showed that kinship declined significantly with distance between male/female pairs. We therefore used the residuals of the linear regression of kinship against square-root transformed distance for all pairs to compare kinship of parental and non-parental pairs (t-test). This analysis was performed separately for each site and using the pooled data.

We investigated possible biases in our data arising from the fact that we could only assign fathers to half of all offspring (e.g. if dead fathers were less related to the mother than those we sampled). For this, we performed one-way analysis of variance to investigate possible variation in kinship among age cohorts of offspring (seedlings, young and old juveniles, immature trees) and also correlated kinship of offspring against height of the fathers (as a proxy for age).

All analyses were performed using R version 4.2.2^26^.

## Results

Using the genotype data, we determined the probable mothers of 269 (55%) of the 493 young *Lodoicea* sampled in four sub-populations on Praslin and Curieuse. We were then able to assign probable fathers to 139 (52%) of these offspring. Thus, for 28% of the offspring sampled, both a probable father and mother could be assigned, with this percentage varying among the 16 clusters. Maternal assignment rates were lower than we had expected, given that we deliberately sampled clusters of offspring growing close to a probable mother tree. This was probably because some clusters included the offspring of female trees that had already died. The overall assignment rate for fathers corresponded closely with our estimate that about half of father trees were dead. Consistent with the progressive mortality of older fathers, paternal assignment rates declined with increasing age of offspring from 67% for seedlings to 25% for immature trees.

**Figure 2:**
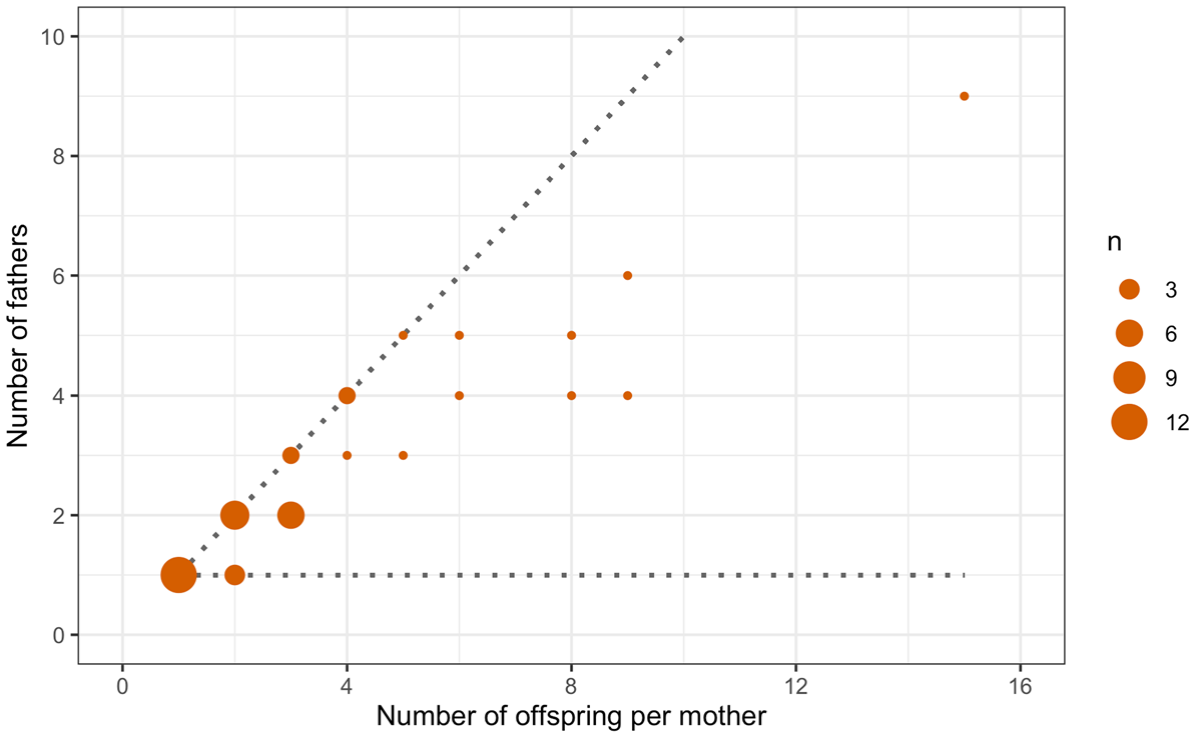
Numbers of fathers plotted against numbers of offspring recorded per mother tree. The diagonal and horizontal dotted lines correspond to 0% and 100% correlated paternity, respectively. Data for 43 mother trees and 139 offspring in four *Lodoicea maldivica* populations.

The 139 offspring in our sample were produced through matings between 43 female trees and 85 male trees, representing 103 parental pairs. The numbers of offspring from individual females varied widely; while over half of females were associated with one or two offspring, there were a few larger clusters of up to 15 offspring from one female (Fig. 2). Correlated paternity (number of full-sib pairs as a proportion of all sibling pairs^27^) for all families with at least two offspring was 0.168, indicating a high level of multiple paternity.

**Figure 3:**
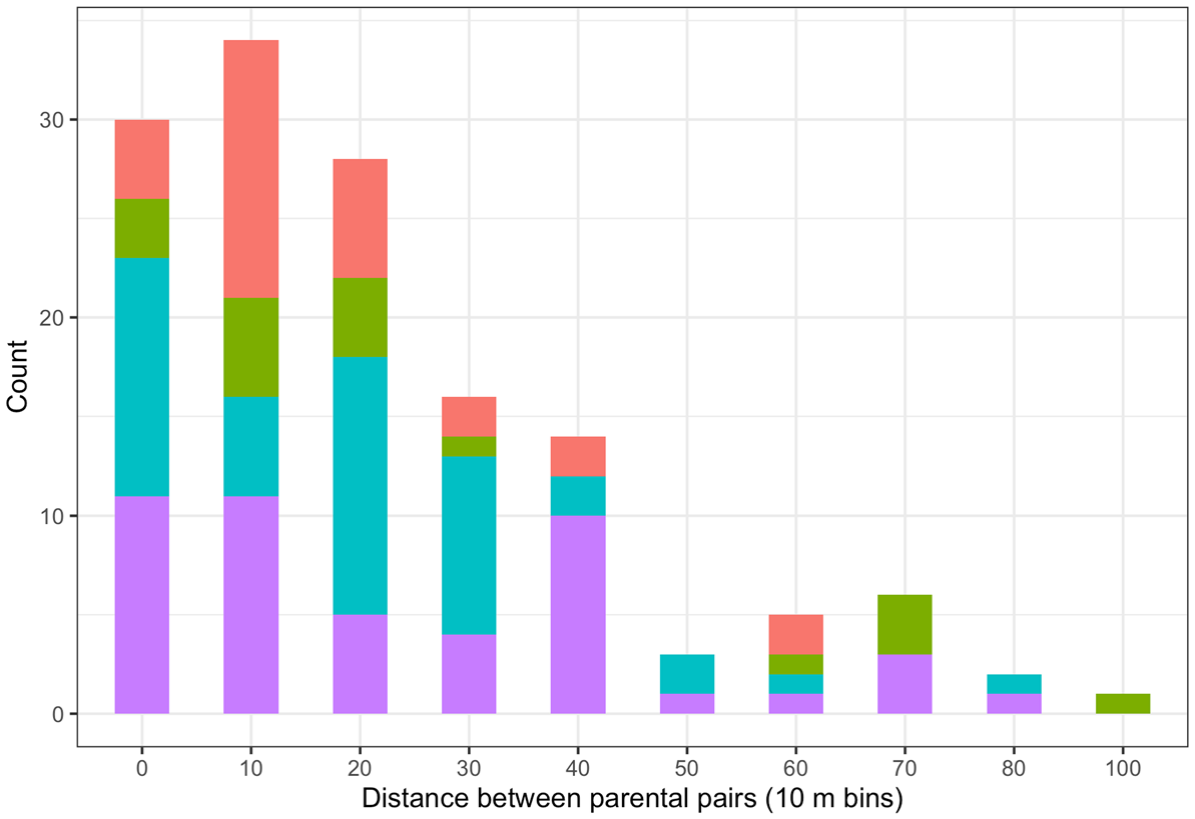
Frequency distribution of realised pollination distances as revealed by 139 offspring genotypes in four *Lodoicea maldivica* sub-populations (purple—VM; blue—FF; green—FP; pink—CU).

The mean distance between mothers and fathers, calculated from the distances between the 139 parental pairs, was 26.8 m, with no significant difference in these distances for male and female offspring (t = -0.778, df = 136, P = 0.438). Two thirds of parent pairs occurred over distances of less than 30 m and the longest distance recorded was 103 m (Fig. 3).

We investigated patterns in Loiselle’s kinship coefficient (F^24^) using (a) all possible pairs of male and female trees within each sub-population (Appendix 2), and (b) pairs with assigned mothers only (Appendix 3). In both cases, the data for the 16 clusters were then aggregated. In the full dataset, kinship ranged between F = − 0.340 and 0.590, with an overall mean of F = 0.0210 (± 0.105 SD; n = 5946). The more restricted dataset produced similar kinship levels at F = − 0.276 − 0.590 and a mean of F = 0.0211 (± 0.104 SD; n = 2485). For subsequent analyses we used the dataset for assigned mothers only, and duplicate parental pairs of more than one offspring were retained in the dataset (Appendix 4). The analysis of kinship for different age cohorts of offspring and for fathers of different ages revealed no significant temporal trends (F = 0.078, df = 2, P > 0.1) that might have biased our results.

**Figure 4:**
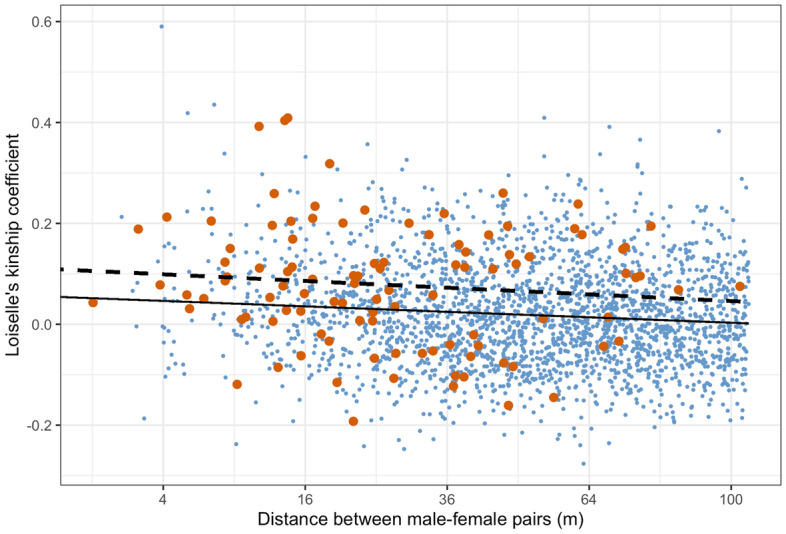
Loiselle’s kinship coefficient (F^24^) for male–female pairs of *Lodoicea maldivica* plotted against square root of distance: blue points—all possible non-parental pairs of the 43 females assigned as mothers; orange points—parental pairs of 139 offspring. Regression lines for the two groups are also shown (all pairs as continuous line; parental pairs as dashed line).

**Figure 5:**
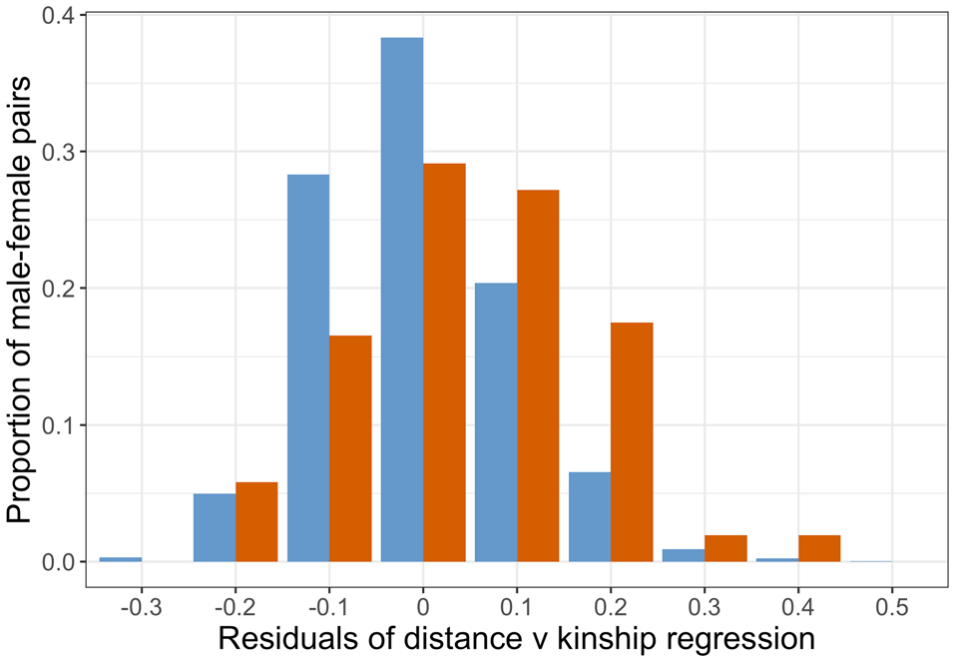
Frequency distributions of residuals from the regression between distance and kinship for all male/female pairs with 48 mother trees: red bars are for 2382 non-parental pairs; blue bars are for 103 parental pairs. Bins are 0.05 ± the values shown.

Linear regressions of kinship against distance (sqrt-transformed) were calculated for 2382 non-parental pairs and 103 parental pairs. For non-parental pairs, there was a significant (R^2^ = 0.011, P < 0.001) negative relationship, indicating that the kinship of male/female pairs declined with distance (Fig. 4). The slope of the regression for 103 parental pairs was not statistically significant (R^2^ = 0.012, P > 0.05). Analysis of covariance revealed no difference in the slopes of the two regressions (P > 0.05), but a highly significant difference in their intercepts (P < 0.001). There was also a significant interaction (P < 0.05) between pair type and site (see Supplementary Tables S1 and S2, respectively, for regression models and ANCOVA). This analysis indicated that, independent of distance, parental pairs were more closely related than expected under random mating, as is also clear from the frequency distribution of regression residuals (Fig. 5). The difference between the mean residuals of parental (0.0365) and non-parental (− 0.001) pairs was highly significant (P < 0.001, Supplementary Table S1). The same analysis performed at a site level (Supplementary Fig. S1) showed a similar pattern at all sites, though the differences between parental and non-parental pairs were only significant at two sites (VM, FF).

## Discussion

### *Dispersal patterns and the genetic structure of *Lodoicea* populations*

A molecular genetic study of remnant *Lodoicea* populations on the islands of Praslin and Curieuse using AFLP’s^28^ found that genetic diversity and heterozygosity were relatively high, while genetic differentiation among populations was very weak. Subsequent work using microsatellites demonstrated intense fine-scale spatial genetic structure (FSGS) and highly significant inbreeding coefficients in these natural populations (F_IS_^17^). The results presented here about the species’ breeding system help in interpreting the genetic structure of *Lodoicea* populations, including some potentially paradoxical findings in the two previous studies.

Intense FSGS and high biparental inbreeding in natural *Lodoicea* populations can be explained by predominantly short-distance dispersal of both pollen and seeds. In a previous study, we found that mean seed dispersal was 8.7 m, with most offspring growing in dense clusters close to the mother trees^17^. Only on steep slopes did seeds occasionally slide longer distances over the forest floor and establish up to 100 m from the mother. Here, we show that mean pollen dispersal (inferred from the distance between parental pairs) is also mainly over short distances (mean 26.8 m), though sometimes occurs over distances up to 103 m. A few fathers may have grown beyond the maximum sampling distance (105 m), but—to judge from paternal assignment rates and mainly short pollen dispersal distances (Fig. 3)—this number was probably small. Another factor that could account for the intense FSGS in *Lodoicea* populations is outbreeding depression, since this would tend to reduce mating success over longer distances; this point is discussed in the next section.

Several factors may explain the apparent paradox of high inbreeding and FSGS coupled with high genetic diversity and weak between-population differentiation. The first is dioecy, which ensures that inbreeding is never as intense as in some monoecious species. On the other hand, spatial genetic structure in populations of dioecious species also tends to be much more strongly developed than in monoecious and hermaphrodite species, as has also been shown within the genus *Ficus,* among others^29,30^. Second, despite short mating distances, correlated paternity as a measure of ‘mate monopolization’^27^ was low, meaning that female trees tended to be pollinated by several males. Third, female fecundity in the study area was typically very low, with trees producing an average of about 1.2 seeds per year^18^. This means that the plants in the larger clusters were the products of matings over extended periods (up to 80 years), during which the surrounding pollination landscape would probably have changed considerably. Fourth, unlike other species of Borasseae, most *Lodoicea* fruits produce only a single seed. It has been suggested that a reduction of ovule number to one per fruit is an evolutionary trend that reduces sibling rivalry^31^, which would be especially significant for a tree with very limited seed dispersal^18^. Finally, it is possible that matings occasionally occur over much longer distances than we could detect. Male *Lodoicea* are on average longer lived than females, sometimes growing to a height of over 30 m, while females rarely reach more than 20 m. Despite possible outbreeding depression, even very rare pollinations over long distances from long-lived emergent trees might be sufficient to maintain a high degree of genetic uniformity across the species’ range.

### Absence of inbreeding depression

Kinship analyses showed that the likelihood of a male–female pair producing offspring was influenced by the pair’s genetic relatedness. After controlling for distance and site (Supplementary Table S2), parental pairs were significantly (P < 0.001) more related than expected on the basis of random mating. The significant interaction between kinship and site (P < 0.05) probably reflects differences in site history, with CU having been far more degraded by fire and clearance than VM. In contrast, there was no evidence that matings between closely related parents were less common than expected under random mating (Figs. 5, Fig. S1), suggesting that inbreeding depression was not an important effect due to habitat degradation. Clearly, experimental studies involving hand pollination would be needed to confirm this conclusion.

The apparent absence of inbreeding depression could be due to the purging of deleterious alleles, as has been suggested for other species in which inbreeding is normal (e.g. in naturally small or isolated populations^5^). Furthermore, as a dioecious species, *Lodoicea* is protected from the extreme inbreeding that can occur in selfing populations. Modelling studies indicate that lethal mutations are more efficiently purged through sib-mating than through selfing^32^. In the oil palm *Elaeis guineensis,* for example, selfing led to a marked inbreeding depression, while sib-crossing not only had a less depressive effect, but was sometimes even superior to outcrossing^33^. This is not to suggest that *Lodoicea* is immune to the effects of inbreeding, however; high levels of even biparental inbreeding could be associated with a gradual increase in genetic load, with negative consequences in the long term^1,6^.

There have been few other studies of dioecious species with restricted pollen dispersal with which our data can be compared, and some of these are ecologically very different (e.g. the submersed aquatic plant *Vallisneria americana*^34^ and the seagrass *Thalassia testudinum*^35^). A study of pollen dispersal and genetic diversity in the dioecious rainforest tree *Fontainea picrosperma* in Queensland, Australia^36^ found mean pollen dispersal distances similar to those for *Lodoicea*, with 63% of seeds sired by male trees located within 30 m of the mother. Levels of inbreeding were negligible, however, suggesting that long-distance gene flow was sufficient to maintain genetic diversity at a stable level across the species’ range. The most comparable study to ours concerns seed and pollen dispersal and spatial genetic structure in the large-seeded dioecious palm *Phytelephas aequatorialis*^37^. In their study site in Ecuador, the authors detected a strongly developed FSGS over distances up to 35 m and inferred mean dispersal distances of 35 m and 68 m for seeds and pollen, respectively. They concluded that the FSGS was maintained mainly by limited seed dispersal, while more extensive pollen dispersal was sufficient to counteract inbreeding.

### Significance of outbreeding depression

Cases of outbreeding depression within populations have often been attributed to the break-up of allelic combinations that provide local adaptation within a heterogeneous environment^11,38,39^, though other factors have also been proposed. For example, mating individuals of the African rainforest tree *Entandrophragma cylindricum* (Meliaceae) were more closely related than expected from pollen dispersal distances^40^, which the authors suggested could be due to genetic influences upon flowering phenology, with closely related individuals showing more synchronous flowering, or to the behaviour of pollinators. These possibilities seem unlikely in the case of *Lodoicea*. Not only is there no evidence for the kind of site heterogeneity, for example in soil conditions or pollinator community, that might produce such an effect, but the outbreeding probability was similar across all distances (Fig. 4). A more likely mechanism is that outbreeding tends to break up favourable allelic combinations associated with seed production. Previous work has shown that the evolution of a huge seed in *Lodoicea* entailed many morphological and physiological adjustments, related in particular to the acquisition of the nutrients needed for making seeds and the mechanical strength needed for bearing them^41^. Given the genetic complexity of seed development^42^, it is possible that many genetic innovations were necessary to produce the seed itself. Seed size is to a large extent determined by the amount of triploid endosperm produced, which depends upon the interplay between the two parental genomes^43^. In fact, *Lodoicea* is far more variable than most plants in the shape and size of its seeds, with fresh weights of individual seeds ranging from as little as 1.5 kg up to 18 kg^44^. It is also variable in the number of seeds produced per fruit, with most fruits containing one seed, but with some containing two and even three seeds. How much of this variability is genetically based is unknown, but natural populations probably contain considerable variation in genes influencing seed size, and could also influence phenology leading to specific parent pair mate choices.

Support for this idea comes from the frequent occurrence of non-viable abnormal fruits, which develop for a year or more but fail to produce any endosperm. In a survey by^44^, abnormal fruits accounted for over half of all developing fruits, being especially common in degraded habitat. Fruit abortion and lower fruit numbers associated have also been linked to both inbreeding and outbreeding in the macaw palm *Acrocomia aculeata*^45^. In the case of *Lodoicea*, the probability of bearing abnormal fruits increased with distance from the nearest male, suggesting that they were produced under conditions of pollen limitation. In contrast, they never developed when mixed, fresh pollen was used in manual pollinations (Terence Payet pers. comm., SIF). This suggests that there could be a dosage effect: when a large number of pollen grains are transferred to the stigma of a distant tree through hand pollination, the chance that one of them will be of a compatible genotype is greater than when only a few grains are transferred by a natural pollinator that requires closed forest to ensure high pollination success^44^. However, we cannot disregard potentially heritable differences also in flower attractants, which we could not determine with our study.

### Implications for management

Our results have implications for management. Local populations of *Lodoicea* exhibit a strongly developed fine-grained genetic structure, and the possible ecological or genetic consequences of disrupting this structure are unknown. In the past, planting schemes aimed at restoring populations have sometimes transferred seeds over long distances. Given the indications that outbreeding depression could operate, seeds should be allowed to grow as closely as possible to where they fall or are collected. For the same reason, hand pollination should usually be performed using a broad range of pollen donors to allow maternal selection of the best allelic combinations. It is worth adding that hand pollinations provide a valuable opportunity to learn more about the breeding system (e.g. how distance between parental pairs affects reproductive success) and it would be useful to record such pollinations, and the subsequent seed production.

The genetic structure of *Lodoicea* is presumably maintained by pollinators that transfer pollen over mainly short distances. Various pollination mechanisms have been proposed, including wind, flies, slugs and trigonid bees^46^, but a recent study suggests that the most important pollinators in natural forest are endemic geckos (SIF, unpublished data). It seems likely that the movement patterns of geckos within closed forest help maintain the high level of multiple paternity that we recorded. If so, the absence of geckos could explain the low seed set and high proportion of abnormal fruits often to be observed in isolated plants^44^. Maintaining a healthy population of endemic geckos should therefore be a high management priority. In addition, we suggest that any planting schemes or habitat restoration should use seeds collected in the same area over time.

In conclusion, we suggest that conservationists should take a more nuanced view of inbreeding and its significance for threatened populations. While it poses a severe threat for many plant species, especially those that formerly occurred in large, unfragmented populations, it may be less critical for species that are prone to limited gene flow, as demonstrated here for *Lodoicea*.

### Supplementary Information

Supplementary Information 1.

Supplementary Information 2.

## Data Availability

Datasets can be found in Supplementary Information Appendices 1–4. We have no sequence data to deposit, however, we do provide our microsatellite genotypes as Appendix 1.
