# Supplementary material for: Mate-choice for close kin is associated with improved offspring survival in Lodoicea maldivica, the largest-seeded plant in the world
Source: Sci Rep. 2023 Sep 18;13:15305. doi: 10.1038/s41598-023-41419-4 (PMC10507110; doi:10.1038/s41598-023-41419-4)
Supplement: Supplementary file 2 — Supplementary Information 2. [file 41598_2023_41419_MOESM2_ESM.pdf]

Supplementary Materials

Article title: Mate-choice for close kin is associated with improved offspring survival in *Lodoicea maldivica*, the largest-seeded plant in the world

Authors: Emma J Morgan, Christopher N Kaiser-Bunbury, Peter J Edwards, Frauke Fleischer-Dogley, Chris J Kettle

The following Supplementary Materials are available for this article:

Table S1 Relationship between kinship and distance for (a) parental pairs and (b) all pairs with the assigned mothers.

a) Parental pairs (N= 103)

Predictor	Estimate	SE	*t*	P
Intercept	0.112	0.035	3.23	0.0017
Distance (sqrt-transformed)	-0.0066	0.0063	-1.06	0.292

b) Non-parental pairs (N= 2382)

Predictor	Estimate	SE	*t*	P
Intercept	0.057	0.0081	7.09	1.81e-12
Distance (sqrt-transformed)	-0.0054	0.0011	-4.94	8.26e-07

Table S2 ANCOVA table showing the significance of the difference in kinship between parental and non-parental pairs (for assigned mothers only) whilst controlling for distance (sqrt-transformed) and site.

Predictor	DF	*Mean Sq*	*F*	P
Parental/non-parental pairs (pairs)	1	0.3442	32.86	1.11e-08 ***
Sqrt_distance	1	0.2681	25.59	4.52e-07***
Site	3	0.0102	0.972	0.4047
Pairs: sqrt_distance	1	0.0007	0.071	0.7901
Pairs: site	3	0.0322	3.079	0.0265 *
Residuals	2475	0.0105		

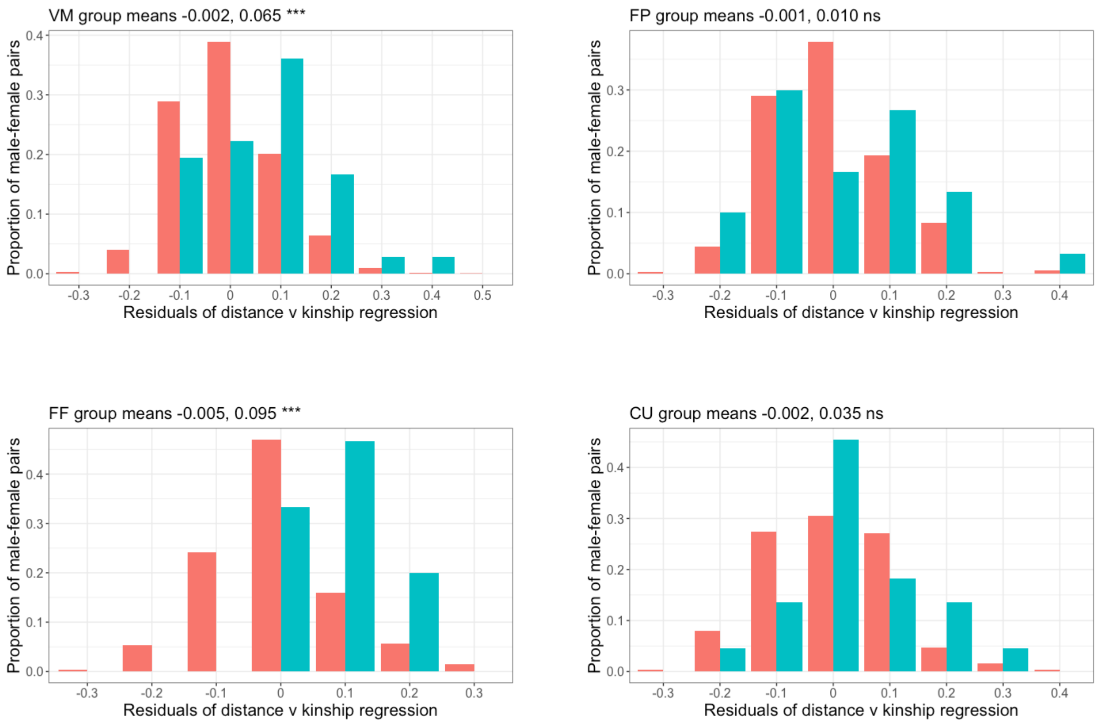

Figure S1. Frequency distributions of residuals from the regression between distance and kinship for all male/female pairs with mother trees at four sites: red bars are for non-parental pairs; blue bars are for parental pairs. Bins are 0.05 ± the values shown. The titles show mean values for each group and the significance of the differences (t-test: ns - not significant, ** - P = 0.01, *** - P = 0.001).
